# Typology of psychiatric emergency services in the United Kingdom: a narrative literature review

**DOI:** 10.1186/s12888-020-02983-5

**Published:** 2020-12-10

**Authors:** Opeyemi Odejimi, Dhruba Bagchi, George Tadros

**Affiliations:** 1 Urgent Care Pathway, Birmingham and Solihull Mental Health Foundation Trust, Birmingham, UK; 2 Aston Medical School, Aston University, Birmingham, UK

**Keywords:** Psychiatric emergency services, Crisis intervention, Mental health services, Mental health crisis, United Kingdom, Literature review

## Abstract

**Background:**

Mental health crisis requiring emergency access to psychiatric service can occur at any time. Psychiatric Emergency Service (PES) is described as one that provides an immediate response to an individual in crisis within the first 24 h. Presently, several types of PESs are available in the United Kingdom (UK) with the aim of providing prompt and effective assessment and management of patients. Therefore, this study aims to provide a detailed narrative literature review of the various types of Psychiatric Emergency Service (PES) currently available in the UK.

**Method:**

Electronic search of five key databases (MEDLINE, PsychINFO, EMBASE, AMED and PUBMED) was conducted. Studies were included if it described a mental health service in the UK that provides immediate response in mental health crisis within the first 24 h. Excluded studies did not describe a PES, non-English, and were not conducted in UK.

**Results:**

Nine types of PESs were found. Amongst the 9 services, more papers described crisis resolution home treatment. Majority of the papers reported services within England than other countries within the UK.

**Conclusion:**

All types of PESs were described as beneficial, particularly to mental health service users, but not without some shortcomings. There is a need to continue carrying out methodological research that evaluate impact, cost-effectiveness as well as identify methods of optimising the beneficial outcomes of the various types of PESs. This may help inform researchers, policy makers and commissioners, service users and carers, service providers and many more on how to ensure current and future PESs meet the needs as well as aid recovery during crisis.

**Supplementary Information:**

The online version contains supplementary material available at 10.1186/s12888-020-02983-5.

## Background

The number of individuals seeking mental health care continues to increase in the United Kingdom (UK). It is now estimated that 1 in 6 individual have one form of mental health problem [1]. Mental health services have continued to evolve over the years, with the first notable change occurring in the 1950s after the closure of asylums and the rise in psychopharmacology [2–4].

The UK has three main kinds of mental health services: routine, urgent and emergency [5]. Routine services are neither urgent nor require emergency intervention. Urgent services are meant for people who require urgent and timely face to face intervention but not immediately life-threatening. Emergency services are delivered to individuals who are deemed to be in a critical situation that is life-threatening to the individual or others and requires an immediate response.

However, the term urgent and emergency mental health services are often used interchangeably and they describe services that respond to “mental health crisis”. According to the National Health Service (NHS) [6] mental health crisis is a situation when an individual experience “sudden deterioration of an existing mental health problem, or are experiencing problems for the first time”.

In this study, Psychiatric Emergency Service (PES) which could also be referred to as mental health emergency service is described as one that provides an immediate response to an individual in crisis within the first 24 h. The aim of these kind of services is to provide assessment, management and in some cases treatment and/or referral for an individual in crisis [7]. In the UK, these services are also called Crisis Intervention Services. In most part of the UK, crisis services are available 24 h a day, 7 days a week. Through the years, economic and political influences have resulted in changes in the pathway and specification of PESs. This has subsequently led to many reforms within the NHS [4].

There have been reviews done on individual kind of PESs [8–11], however, none has been done on all forms of PESs currently existing in the UK. The purpose of this review is to provide a detailed coverage of all forms of PES in the UK. To the best of our knowledge, this is the first review to describe all types of PES currently available within the UK. Thus, the aim of this paper is to provide a detailed literature review of the different types of PES currently available within the UK. Each type of PES will be described in terms of its structure, process, and outcomes. Furthermore, methodological qualities of current studies and the relevant issues to be investigated will be discussed.

## Research questions

- What is the current scope of psychiatric Emergency Services in the UK?
- What are the relevant issues impacting various types of PES in the UK?
- What are the current gaps in research about PESs in the UK?

## Methods

### Search strategy

Electronic search was conducted on October 24th 2018 and November 2nd 2018, on five key databases (MEDLINE, PsychINFO, EMBASE, AMED and PUBMED). The search terms used in this study is detailed in Table 1 below.

**Table 1:** Search terms

emergenc* OR Crisis OR crises OR Urgent Psychiatr* or mental health Unit* OR center* OR centre* OR model* OR service* United Kingdom OR Scotland OR England OR Great Britain Wales NOT south Ireland NOT republic “Street Triage” OR “section 136” OR “section 297” OR “place of safety” Liaison AND “emergency department” OR “Accident and Emergency” Assessment OR decision OR Triage	

### Inclusion and exclusion

Studies written in English and conducted within the United Kingdom (UK) which described a form of PES that fit the definition of PES were included in this review. We describe Psychiatric Emergency Service (PES) as a mental health service that provides an immediate response to an individual in crisis within the first 24 h. Search was not limited by years and this is to help have a comprehensive overview as well as show changes over time of the various models of PES. Studies which did not meet any of the criteria detailed above were excluded.

### Search outcome

**Fig. 1:**
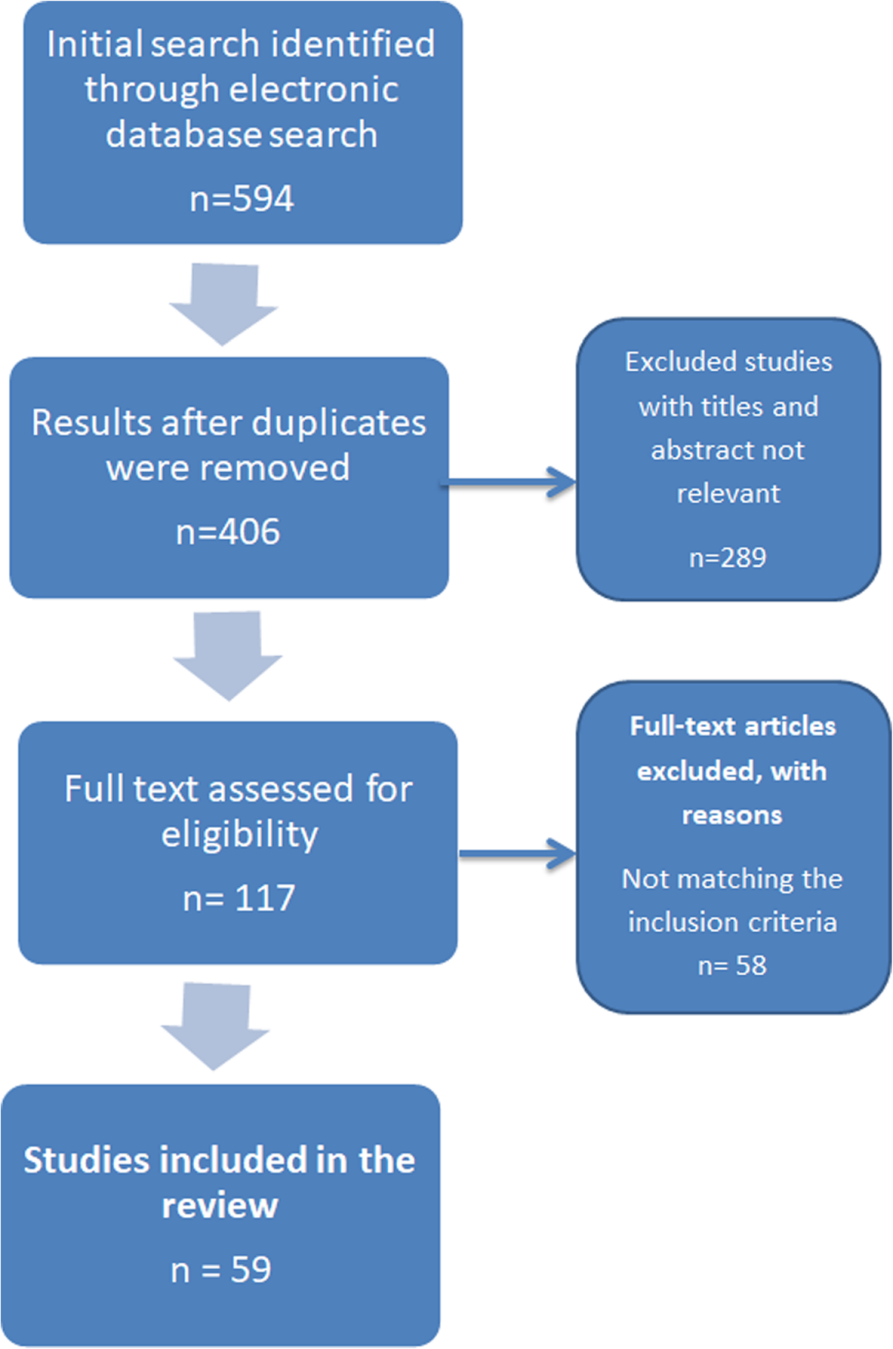
Search outcome

Figure 1 provides details of the search outcome. The literature search from the five databases revealed 594 papers; this was reduced to 406 after duplicates were removed. Of the 406 papers, 298 were excluded after screening the title and abstract resulting in 117 papers. Full text of the 117 papers were retrieved and screened against the inclusion criteria. 59 studies were included in this study. Papers were excluded if they did not the inclusion criteria.

### Data extraction

Two reviewers performed data extraction and any disagreements were resolved by discussion. See Additional file 1 for list of included studies. The following information was extracted: first author’s name, year of publication, city, type of PES described, methodology used, country within the UK the study was conducted and the study aim.

### Methodological qualities

**Fig. 2:**
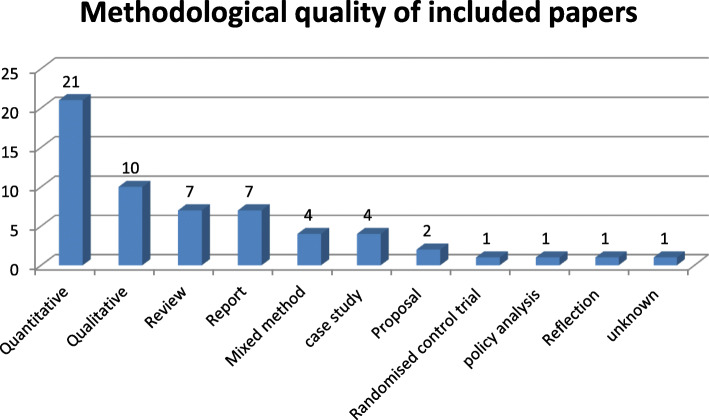
Methodological qualities of included papers

Papers found were of diverse methodology. Majority of the papers were quantitative studies, mostly retrospective surveys to evaluate the effectiveness of the service. Both systematic and literature reviews are referred to as ‘*review’*. We classed newspaper report, commentaries or editorials as ‘*reports’*. Furthermore, *‘case studies’* are described as detailed description of a particular service or review of case notes, while proposal and protocols were called ‘*proposal’* On some occasions, a second paper was then published detailing the impact of the service. We only found one Randomised Control Trials (RCT) [12] and a proposal for a RCT [13] to be carried out. However, the full study of the protocol was not found at the time this review was conducted. Figure 2 below shows the methodological qualities of included studies.

## Results

### Typology of psychiatric emergency services

In total, nine type of PESs were identified. They are: crisis resolution and home treatment, place of safety, police officer intervention, psychiatric assessment unit, mental health liaison services, integrated service and voluntary sector. There were more papers describing Crisis Resolution Home Treatment (CRHT) services than the others. Seven papers described both Police Officer Intervention (POI) and Place of Safety (POS) in their papers. Table 2 below gives an illustration of the numbers of papers describing each kind of PES identified.

**Table 2:** Types of Psychiatric Emergency Service identified

Type of Psychiatric Emergency Services identified	Number of studies describing the service
Crisis Resolution and Home Treatment (CRHT)	15
Place of Safety (POS)	5
Police Officer Intervention (POI)	4
Both POS and POI	7
Street Triage	3
Psychiatric Assessment Unit (PAU)	11
Mental Health Liaison Services (MHLS)	3
Integrated service (IS)	8
Voluntary Sector (VS)	1
Crisis House	2

### Country of origin of included studies

Majority of the papers reported services in England. Six papers described PES both in England and Wales and only one was exclusively about a service in Wales. Three papers were found in Scotland, of which two were a longitudinal study, thus, classified as one. This is because the second study [14] was carried out to compare findings from the first study [15] and both studies had the same aim. Furthermore, only one paper reported a service in Northern Ireland.

## Discussion

Each type of PES will be described in terms of its structure, process, shortfalls and its perceived benefits.

### Police officer intervention (poi) and place of safety (POS)

Police officers in the UK have the power to detain an individual considered to be in mental health crisis in a public place in order to keep the individual and/or members of the public safe [16]. The term used to refer to this power, code of practice and duration of detention varies across the 4 countries in the UK [16–18].

The Police Officers Intervention (POI) services aims to keep individuals in mental health crisis detained in a safe environment in order to complete a comprehensive assessment by a mental health professional [18–20]. This safe environment is often referred to as “Place of Safety” (POS) and it could be a police station, hospital, residential home or mental health institution. Some authors refer to POS as S136 suites and some studies use both terms interchangeably [8, 20, 21]. This explains why it is quite common to find a paper that describes both services as one. Nearly all papers describing POI and/or POS were from England, three were from both England and Wales, with only one from Scotland.

Profile of the detainees is similar across the 4 countries as they often have previous mental health history, suicidal intent and/or self-harm with underline diagnosis of schizophrenia, drugs and substance induced psychosis, alcohol and drug misuse, mania and personality disorder [18, 19, 22]. Reported socio-economic status and demographics of individual who utilise POI and POS are mostly those with no fixed abode, unemployed, men, black ethnicity [23, 24]. The behaviour leading to arrest includes threatening or actual violence or self-harm, causing disturbances, drugs or alcohol misuse [23].

A literature review by Apakama [8] identified four kinds of POS: police custody, A & E, Psychiatric Unit, and Intermediate Care Facilities’ (section 136 suites). This author concluded that none of the POS described can be considered the most appropriate for all groups of patient who are detained under S136.

Over the years, there have been controversies about POI and POS with studies highlighting its ethical and moral concerns. This has been attributed to inconsistencies in police officers judgement about detention, lack of training of police officers in mental health, conveying detainees in police vehicles and the use of police stations as POS [18, 19, 25].

In the light of this, it has been strongly recommended that police station should only be used in exceptional circumstances [26]. It is hoped that this will allow patients feel less criminalised and mental health professionals carry out assessment and management promptly. However, police stations are still in use as POS based on pragmatic reasons such as: absence/shortage of spaces at designated POS, shortage of mental health staff, person displaying or with previous history of violence and alcohol intoxication [20].

Overall, the use of POI to manage mental health crisis in public places is important yet not without complexities. Likewise, having a designated POS suite or centre that caters for the needs of patients and equally acceptable to detainees, mental health professionals, and the police should be the ideal, but this might be far reaching. Thus, a systematic review of current POI and POS model within the UK to ascertain its effectiveness and cost-effectiveness is needed.

### Street triage (ST)

Street Triage (ST) is a collaborative mental health service by the police and mental health professionals delivered to prevent unnecessary detention of an individual in mental health crisis [27, 28]. These services have been developed in response to reviews and reports about mismanagement of individuals in crisis using POI and POS [28]. For instance, the Bradley report [29] and the Crisis Concordant [30] called for a more collaborative practice between organisations to work in partnership in order to adequately improve support and treatment for individuals in mental health crisis.

As a result of the close link between the criminal justice system and mental health services, it is quite common to find a paper that describes how POI services was initially used and then patient transferred to street triage. Nearly all papers on ST were from England and only one [31] did not specify the region where the study was conducted. We found three models of STs described and they are: specialist police officers response, specialist mental health professional response and a telephone triaging collaborative approach [31, 32].

Most of the Street Triage in the UK is the specialist mental health professionals’ response type where a mental health professional (usually a nurse) is stationed within the police control rooms with the aim of referring an individual in crisis to existing mental health services [27, 31, 33]. The specialist police officer response model is one in which the police officer have received mental health training to respond to mental health crisis [31]. The telephone collaborative approach is one in which a mental health professional is available on the telephone to offer advice or give information to patrol police officers [31].

All three approaches have recorded positive outcomes. These include: significant reduction in the use of POI and admission from POI detention, positive service users feedback, less police time and resources, improved communication and understanding between the police and mental health services and improved care pathway for dealing with mental health crisis [27, 28, 31]. It has been identified that the success of ST can be majorly attributed to the expertise of the mental health staff.

The drawback with ST is majorly attributed to staffing arrangements for mental health and police mangers as the current shortage of staff from both services might impact on the effectiveness of ST [31]. Moreover, ST may not be saving money as indicated if service users are not directed to the right services. For example, Heslin [34] study noted that referrals were made to General Practitioners (GP) and the A & E.

Generally, ST is viewed as a PES with many benefits. However, Methodological evaluations of its impact are limited. Moreover, there is a need to conduct longitudinal studies to ascertain its effectiveness in the long run. Furthermore, a comparison of the three models of ST described above can be investigated to identify which of the models are the most effective and cost effective and also help address the drawbacks identified above.

### Mental health liaison service (MHLS)

Mental Health Liaison Service (MHLS) aims to provide assessment and treatment for individuals in hospitals with co-morbid physical and mental illness [35, 36]. MHLS is not a new concept, and has been described in literature since the 1970s [37]. However, the increasing presentation of co-morbid physical and mental illness at A & E and inpatients have further drawn attention to this concept [36, 37]. In fact, Plumridge and Reid [38] stated that 28% of acute inpatients have co-morbid mental and physical illnesses and this number rises to 60% when older patients with delirium and dementia are included. Furthermore, the recognition that health care professionals may not be skilled enough to manage the needs of mental health patients has increased the need for MHLS [36].

We recognise that there are various models of MHLS and many hospitals have taken on board the Royal College of Psychiatrists [8] recommendation about mental health liaison as an essential service needed in all acute hospitals. However, In line with the definition of PES described earlier, only papers describing MHLS that deliver urgent and emergency care and support to individuals in crisis within 24 h of presenting in the A & E were selected. Nearly all papers about MHLS are from England, with only one from Wales and one from an unspecified region of UK. In one study, MHLS was referred to as Rapid Assessment interface and Discharge service [35].

Reported beneficial outcomes of MHLS are: reduction in patients’ readmission and length of stay in hospital, better patient satisfaction, reduce length of time at A & E, overall, saving cost to the local hospital [36, 39]. Little has been reported with regards to its drawbacks as most of the studies were cross-sectional studies that focused more on its beneficial impact. Thus, there is a need to conduct longitudinal studies that highlights both beneficial and negative impact of this service. This will result in a more balanced view of MHLS and also help recognise areas that require improvement with the aim of optimising its beneficial outcomes.

### Voluntary sector and crisis house

Voluntary Sector (VS) also known as the third sector, non-profit, or non-governmental. They provide a wide range of crisis support services which includes: peer/group support, crisis café, helplines, crisis house and other forms of alternative to inpatient care [40]. VS mental health services provision is often viewed as complementary to the already existing statutory ones [40]. MIND [41] made clear that they close the gap brought about by failures in service provision by statutory organisations. For instance, they provide better access and more service user-led services that reach out to Black and Ethnic Minorities and others who are hard to reach [40].

In this review three papers were identified of which all were from England. Also, one was a proposal to evaluate voluntary sector provision of crisis services and the other two were studies on crisis houses delivered by statutory organisations. The authors recognise that some crisis houses are also provided by statutory organisation. However, in this study both have been considered together. This is because on some occasion crisis house serve as alternative residential arrangement to inpatient care for individuals in crisis and are often provided by VS [40].

It has been reported that mental health patients prefer crisis house to hospital based services because they perceive crisis houses as less stigmatising and institutionalised [42, 43]. Moreover, MILMIS project Group [44] stated that crisis houses serve as a better option to inpatient service especially in areas noted for hospital bed shortage. However, there are limitations to individuals who can be admitted to crisis house, these include, persons detained under the Mental Health Act, as well as those regarded as being of violent behaviour, or individuals misusing drugs or alcohol which require detoxification [42, 43].

It has been pointed out that the profile of individuals using voluntary services and crisis house when compared with statutory services are very similar, yet, VS does not have as much recognition in research. Usually, larger voluntary organisations profile indicates their active involvement in crisis management; however, only few researches demonstrate and document the extent crisis support services are provided by smaller VS organisations [40]. Hence, more methodological evaluation of VS provision of crisis services need to be carried out and it will be suggested that all stakeholders views such as the service users, staff and management be explored to have a holistic perception.

### Crisis resolution home treatment (CRHT)

The Crisis Resolution Home Treatment (CRHT) service serves as a great alternative to inpatient care for individuals in mental health crisis with the aim of delivering rapid assessment, support and care for individuals in the confines of their home and family [9, 10, 13, 45]. CRHT is one of the popular crisis services in England due to the mandatory declaration under the NHS plan in 2002 [46]. This is no longer a mandatory service but it remains an essential service with guidelines and reports strongly recommending it [47].

This kind of service has been called various names. For instance: ‘crisis resolution’, ‘crisis assessment and treatment’ as well as ‘intensive home treatment’ [45]. Nevertheless, Morgan and Hunte [48] made clear that CRHT remains a more acceptable name and the one mostly used in public report. All but two papers identified in this review about CRHT were from England, with one from an unspecified region of UK [49] and the other a collaborative effort between England and Norway [10].

The CRHT ideally provides 24 h, 7 days a week, rapid emergency assessment and also review patients daily, with the intention of minimising disruption to patients’ daily lives over a period of 4–6 weeks [10, 50, 51]. However, various models of CRHT currently exist, but most CRHT are run by multidisciplinary team offering home-based services. It should be noted that there are variations in the structure of CRHT in different regions of the UK and these variations are based on local needs of patients within each region [10]. Reported outcome include: reduce hospital admission subsequently saving cost, and better user satisfaction [9, 10, 12, 52]. The success of this service has been attributed to its being a mobile service, home based and the strong emphasis on family and social network helping in the recovery process [10].

Nonetheless, one study indicated no reduction in hospital admission following its implementation [53]. These authors claimed that there was already a more proactive measure of reducing admission rate prior to conducting their study. Studies that have reported the negative impacts of CRHT are very limited [10]. Such researches are valuable as they help develop strategies on how to optimise its beneficial outcomes and not to demean CRHT. Therefore, research on all possible outcomes of CRHT based on the various models being used is highly recommended. This will help policy makers identified evidence based strategy that is effective and cost-effective.

### Psychiatric assessment unit (PAU)

Psychiatric Assessment Unit (PAU) is one of the models of PES that emerged based on the need to provide comprehensive assessment to individuals in crisis [54, 55]. The PAU is a dedicated 24-h mental health urgent assessment unit aimed at providing proper triaging for individuals in crisis. It is mostly run by mental health nurses and overseen by consultant psychiatrist. It prevents unnecessary hospitalisation and may offer comfort measures (food, shelter, shower, bed) and treatment usually up to 72 h [55, 56].

It has been documented that many of the referrals received are from A & E because mental health patients are more likely to present at the A & E during crisis [57, 58]. There is evidence that the A & E is not an ideal environment for individuals in crisis and studies have highlighted these drawbacks to patients, staff and facilities [57, 58]. Reported drawbacks include: increased level of stress/crisis of patients, negative staff attitude, increased burden on resources of the facilities, inadequate or improper assessment of patients and many more.

Research on this type of service is scarce. We found only 2 relevant papers via electronic database search, of which one was a conference paper and the other an editorial. When we carried out online search, we discovered three NHS Trusts currently running this kind of service however, all three used different names to describe their service, such as: ‘mental health assessment unit’, ‘psychiatric decision unit’ and urgent psychiatric assessment service’.

Furthermore, online internet search a poster by a NHS Trust which was later developed into a full article [59] and an audit by Ul Haq et al. [55] revealed similar results. They both reported a significant in-patient admission reduction and also lesser burden on the already stretched A & E department. However both studies were only carried out for a short period of one and six month for Trethewey et al. [59] and Ul Haq et al. [55] respectively. Moreover, the views of stakeholders were not taken into cognisance. Thus, there is a need for more comprehensive longitudinal evaluation of this kind of service that takes into cognisance all stakeholders views.

### Integrated services

An integrated service is one which incorporates two or more PES services in order to provide a holistic crisis intervention. Services describing POI and POS or both Street triage and POS in a single paper were not classed as an integrated service. This is because when individuals are seen by the police via POI or the Street Triage services, they are often taken to a POS; hence, there is a greater tendency to describe these types of services in a single paper.

We found 8 papers describing more than one kind PES; however, they do not meet our description of an integrated service. This is because majority of the papers provided results of surveys or reviews of more than one service without the services necessarily integrated. There were more papers from England. Papers were also found from Northern Ireland and Scotland and one made comparison of available PES in UK and globally.

One paper described integration of two or more services [60]; however, it cannot be classed as an integrated PES. This is because this paper discussed three services, inpatient beds, CRHT, and Acute Day Care. Nevertheless, neither inpatient beds nor Acute Day care can be classed as a PES, because both type of services do not necessarily render crisis services in the first 24 h as indicated in our definition of PES earlier.

Hence, there is a need to identify studies that incorporate at least two or more PES described above. Moreover, those services should be evaluated to see if it is more effective and cost-effective than single PES.

### Limitation

This review identified papers mostly via electronic search of the 5 database and in some cases, online internet search. Other means such as citation scanning, internet sources, reference list scanning and hand searching of journal publication were not explored. Thus, we recognise that there could be more typologies and studies describing PES. Nevertheless, our study yielded a rich result which identified 9 types of PESs in the UK.

## Conclusion

Reported findings of PESs have indicated that all types of PESs are somewhat beneficial particularly to mental health service users, but not without some shortcomings. This review provides the first known attempt to illustrate the wide variation in the provision of PES and also variations within the same model of PES in the UK. Our recommendation for further research is for more methodological evaluation of each PES, we recommend longitudinal studies and possibly randomised control trials to effectively study its impact on all stakeholders as well as its cost effectiveness. Overall, this study has provided a narrative review of PESs in the UK which could be used as a benchmark for further exploration of PES within the UK. Moreover, researchers, policy makers and funders who are seeking knowledge of existing PESs in the UK and next direction in service provision and research may use this study to steer their decisions.

## Supplementary Information

**Additional file 1:** List of included studies

## Data Availability

All data generated during this study are included in this published article and other data supporting findings are available as additional supporting files.

## Abbreviations

PES: Psychiatric Emergency Service
UK: United Kingdom
NHS: National Health Service
CRHT: Crisis Resolution Health Team
POI: Police Officer Intervention
HBPOS: Health Based Place of safety
POS: Place of Safety
IS: Integrated service
MHLS: Mental Health Liaison Service
PAU: Psychiatric Assessment Unit
VS: Voluntary Service
RCT: Randomised Control Trials
ST: Street Triage
A & E: Accident and Emergency
