# Supplementary material for: Typology of psychiatric emergency services in the United Kingdom: a narrative literature review
Source: BMC Psychiatry. 2020 Dec 10;20:587. doi: 10.1186/s12888-020-02983-5 (PMC7727184; doi:10.1186/s12888-020-02983-5)
Supplement: Supplementary file 1 — Additional file 1: List of included studies [file 12888_2020_2983_MOESM1_ESM.docx]

**INCLUDED STUDIES**

*(CRHT*- Crisis Resolution and Home Treatment, POS*- Place of Safety, POI*- Police Officer Intervention, PAU*- Psychiatric Assessment Unit, MHLS*- Mental Health Liaison Services, IS*- Integrated service, VS*- Voluntary Sector, ST*- Street Triage)*

| **S/N** | **Author and year** | **Type of Psychiatric Emergency service** | **Methodology** | **Country** | **Study Aim** |
| --- | --- | --- | --- | --- | --- |
|  | Heslin et al (2016) | POI/POS | Quantitative | England | To determine if street triage is effective at reducing the total number of people with mental health needs detained under section 136, and is associated with cost savings compared to usual police response |
|  | Keown et al (2016) | POI/POS | Quantitative | England | To describe the impact of Street Triage (ST) on the number and rate of Section 136 Mental Health Act (S136) detentions in one NHS Mental Health and Disability Trust |
|  | Greenberg et al (2002) | POI | Quantitative | England | Give details about S136 use especially in a rural area |
|  | Horspool et al (2016) | POI/POS | Qualitative | UK | Peer reviewed assessment of the effectiveness of street triage. Examination of the design and potential impact of two services, along with factors that hindered and facilitated the implementation of the services. |
|  | Apakama (2012) | POI/POS | Review | UK | To review the literature and determine the ideal place for the detention and assessment of these patients and clarify the responsibilities of the staff of the agencies involved in the detention |
|  | Riley et al (2011) | POI | Qualitative | England | Reports on the views of detainees and their carers of their experiences of being detained under Section 136 (S136) of the Mental Health Act 1983. |
|  | Kane et al (2017) | **IS***   - MHLS - ST - CHRT - POI | Review | England and Wales | A reviews of all of this services |
|  | Cole (2014) | ST | Report | England | Describes street triage services |
|  | Walker et al (2018) | MHLS | Quantitative | England | To describe the current provision of hospital based liaison psychiatry services in England, and to determine different models of liaison service that are currently operating in England. |
|  | Opmeer et al (2017) | MHLS | Quantitative | England | To evaluate the impact of an expansion of  liaison psychiatry services (LPS) on patient management, outcomes and treatment costs for emergency department (ED) attendances for self-harm. |
|  | House et al (2018) | MHLS | Mixed methods | England | To investigate the Mechanisms by which liaison mental health services (LMHS) may bring about improved patient and  organisational outcomes are poorly understood |
|  | Keown (2013) | POI/POS | Quantitative | England | To detail changes in the use of place of safety orders in England, including the outcome of these detentions, |
|  | Hampson (2011) | POS | Report | England and Wales | A call to raise standard of section 136 |
|  | Laidlaw et al (2010) | POI | Quantitative | England | Studied the use of Section 136 in a rural area |
|  | Waghorn (2010) | MHLS | Report | England | Describing the impact of having MH liaison nurse at ED |
|  | Menkes and Bendelow (2014) | POI | Qualitative | England and Wales | purpose of  this paper is to examine police attitudes to and criteria for using s136 |
|  | Laidlaw et al (2009)  Pugh and Laidlaw (2016) | POS  POS | case study  Case study | England  England | New POS set up  Running of POS |
|  | Howe et al (2003) | MHLS | Quantitative | England | Survey of MH liaison service |
|  | Sondhi et al (2018) | POI/POS | Qualitative | England | The aim of this study was to describe the views and perceptions of the process for people with lived experience of mental distress who have been detained under section 136 of the Mental Health Act 1983 |
|  | Lowe-Pomsford and Begg (1996) | POS | Case study | England | Description of s136 in a airport environment |
|  | McClimens et al (2017) | MHLS | Review | England | reviews the literature, as well as  outlining the background to, and method of, evaluation of MH liaison team |
|  | Eales et al (2006) | MHLS | Qualitative | England | To determine what is important to stakeholders in a liaison mental healthcare  service |
|  | Morgan and Coleman (2000) | MHLS | Quantitative | Wales | determine what is important to stakeholders in a liaison mental healthcare service |
|  | Callaghan et al (2001) | MHLS | Quantitative | England | To analyse the work of a liaison mental health service at the Accident and Emergency (A & E) department of a hospital |
|  | Ryrie et al (1997) | MHLS | Mixed methods | England | This paper describes a study into the work of two emergency psychiatric nurses (EPN) in a MH liaison Team |
|  | Robert (1997) | MHLS | Review | UK | Describes how mental health liaison nurse role evolved in the UK |
|  | Ulhaq et al (2011) | PAU | Quantitative | England | To evaluate the MH assessment unit |
|  | Agrawal and Murphy (2008) | PAU | Report | England | To describe a Psychiatric assessment unit |
|  | Kirubarajan et al (2018) | ST | Quantitative | England | Information about street triage  Police officers views and experiences |
|  | Lloyds-Evans et al (2018) | CRHT | Quantitative | England | Implementation of Mental health crisis resolution in England |
|  | Newbiggings et al (2017) | VS | Proposal | England | Contribution of voluntary section in delivering mental health crisis services |
|  | Morant et al (2017) | CRHT | Qualitative | England | Stakeholders experiences and views about CRT |
|  | Nelson et al (2016) | CRHT | Qualitative | Unspecified | Stakeholders views about CRHT |
|  | Lloyds-Evans et al (2016) | CRHT | Proposal | England | Proposal to evaluate the impact of CRT on service users experiences of care, service use, staff wellbeing, and team model fidelity |
|  | Paton et al (2016) | **IS***  Available services | Review | United kingdom and more global | Understand the clinical effectiveness and cost-effectiveness of crisis resolution teams |
|  | Morrow et al (2016) | **IS***  Integrated crisis plan –  Mental Health Crisis Service incorporating  inpatient beds, a high fidelity model Crisis Response Home Treatment Team and Acute Day Care facilities | Quantitative | Northern Ireland | Evaluate the effectiveness of the integrated approach |
|  | Wilson-Palmer and Poole (2015) | ST | Case study | England | Describe a model of street triage and state its impact |
|  | Wheeler et al (2015) | CRHT | Review | England | To compare CRT and treatment as usual |
|  | Loader (2014) | CRHT | Policy analysis | England | Critical analysis of policy relating to provision of 24 hour mental health access with a focus on CRHT |
|  | Wise (2014)  Wise (2013) | POS  POS | Report  Report | England and wales  England and Wales | Call for a dedicates suite for section 136 individuals  Call for a place of safety |
|  | Sprtinks (2014) | POS | Report | Unspecified | Call for more urgent crisis care |
|  | Duffin (2014) | **IS***   - POS - crisis resolution team | Report | Unspecified | Call to improve mental health crisis care |
|  | Ferguson et al (2010) | CRHT | Qualitative | England | To understand factors that hinder or facilitate MH CRT |
|  | Sjolie et al (2010) | CRHT | Review | UK and Norway | Explore and systematize the existing knowledge regarding the structure, process, and outcome of crisis resolution and home treatment |
|  | Morton (2010)  Morton (2009) | CRHT  CRHT | Case study | England | explore the relationship between emotion and the experience  of a mental health crisis, in particular how this relationship is understood in crisis resolution services  Nature of crisis in people presenting to the CRT, the range of interventions received by individuals by CRT and staff view of the element of intervention important |
|  | Johnson et al (2010)  Johnson et al (2009) | **IS***  A comparative study on alternatives to in-patients-  A comparative study on alternatives to in-patients- | Mixed methods  Mixed methods | England  England | Comparison of residential alternatives to acute services  To develop a typology of services and describe their distribution and clinical populations |
|  | Lyons et al (2009) | **IS***   - CRHT - respite services | Mixed methods | England | Users and carers definition of crisis, range of crisis services available, resources and intervention that help avoid unnecessary hospital admission |
|  | Khalifeh et al (2009) | CRHT | Qualitative | England | To explore the experiences, treatment preference and needs of mothers with dependent children treated by CRT |
|  | Howard et al (2008) | Crisis House | Quantitative | England | Compare the sociodemographic and clinical characteristics of women admitted in a women crisis house and psychiatric hospitals |
|  | Hopkins and Niemiec (2007)  Hopkins and Niemiec (2006) | CRHT  CRHT | Qualitative  Qualitative | England  England | Perspective of service users on home treatment service  Perspective of service users on home treatment service |
|  | Johnson et al (2005) | CRHT | Quantitative | England | Effectiveness of crisis resolution team |
|  | Dunn (2001) | CRHT | Quantitative | England and Wales | Effects of home treatment on the numbers and circumstances of assessment in accordance with the mental health Act 1983 |
|  | Pullen and Nisbet (2001)  Kehoe and Pullen (1992) | **IS***  A mix of 24 hours services for emergency psychiatric assessment in Scotland  A mix of services in Scotland | Quantitative  Quantitative | Scotland  Scotland | Survey services providing PES  To describe Scotland’s PES |
|  | Killaspy et al (2000) | Crisis House | Quantitative | England | To describe women only crisis house |
|  | Reed (1998) | CRHT | Reflection | England | Discusses the work of the psychiatrist and family therapist to develop crisi intervention |
|  | Huxley and kerfoot (1995) | **IS***  Study of various services | Quantitative | Unspecified | Describes typology of crisis service |
|  | Tufnell et al (1985) | CRHT | Unknown | Unspecified | Describing a new CRHT |
|  | Macaskill et al (2011) | POI/POS | Quantitative | Scotland | To establish the quality of documented information provided by the police; adherence to the protocol; completion rates of documentation; and rates of notification to the Mental Welfare Commission for Scotland, in keeping with the standards set in the Code of Practice of the Mental Health (Care and Treatment) (Scotland) Act 2003. |
|  | Hughes et al (2017)    Tretheway et al (2019) | PAU  PAU | Quantitative (poster)  Quantitative | England  England | To evaluate the activities of the PDU and its  impact on the frequency of ED presentations and inpatient admissions, and to explore patient satisfaction |

**REFERENCES OF INCLUDED STUDIES**

1. Heslin, M. et al. (2016) ‘Decision analytic model exploring the cost and cost-offset implications of street triage’, BMJ Open, 6(2), pp. 1–10. doi: 10.1136/bmjopen-2015-009670.
2. Keown, P. et al. (2016) ‘Too much detention? Street Triage and detentions under Section 136 Mental Health Act in the North-East of England: A descriptive study of the effects of a Street Triage intervention’, *BMJ Open*, 6(11), pp. 1–8. doi: 10.1136/bmjopen-2016-011837.
3. Greenberg, N. et al (2002) A prospective survey of Section 136 in rural England (Devon and Cornwall). *Medicine, Science and the Law*. 42(2). pp. 129-34.
4. Horspool, K., Drabble, S.J. and O’Cathain, A., 2016. Implementing street triage: a qualitative study of collaboration between police and mental health services. *BMC psychiatry*, *16*(1), p.313.
5. Apakama, D. C. (2012) ‘Emergency department as a “place of safety”: Reviewing the use of section 136 of the mental health act 1983 in England’, Medicine, Science and the Law, 52(1), pp. 1–5. doi: 10.1258/msl.2011.010154.
6. Riley, G. et al. (2011). ‘A frightening experience’: detainees’ and carers’ experiences of being detained under Section 136 of the Mental Health Act. *Medicine, Science and the Law*, *51*(3). pp 164-169.
7. Kane, E., Evans, E., & Shokraneh, F. (2017). Effectiveness of current policing-related mental health interventions in England and Wales and Crisis Intervention Teams as a future potential model: a systematic review. *Systematic reviews*, *6*(85). pp 1-7.
8. Cole, E. (2014). Triage teams are a streetwise success story. *Nursing standard (Royal College of Nursing (Great Britain): 1987)*, *29*(9). pp 20-22.
9. Walker, A. et al. (2018). Organisation and delivery of liaison psychiatry services in general hospitals in England: results of a national survey. *BMJ open*, *8*(8), e023091.
10. Opmeer, B. C. et al. (2017). Extending the liaison psychiatry service in a large hospital in the UK: a before and after evaluation of the economic impact and patient care following ED attendances for self-harm. *BMJ open*, *7*(8), e016906.
11. House, A. et al. (2018) A programme theory for liaison mental health services in England. BMC health services research, 18(742). pp 1-11.
12. Keown, P. (2013). Place of safety orders in England: changes in use and outcome, 1984/5 to 2010/11. *The Psychiatrist*, *37*(3). pp 89-93.
13. Hampson, M. (2011) ‘Raising standards in relation to Section 136 of the Mental Health Act 1983’, Advances in Psychiatric Treatment. Cambridge University Press, 17(5), pp. 365–371. doi: 10.1192/apt.bp.110.008250.
14. Laidlaw, J. et al. (2010). The use of Section 136 (Mental Health Act 1983) in Gloucestershire. *Medicine, Science and the Law*, *50*(1). pp 29-33.
15. Waghorn, J. (2010). The impact of basing mental health liaison nurses in an emergency department at night. *Journal of psychiatric and mental health nursing*, *17*(7), 647-650.
16. Menkes, D. B., and Bendelow, G. A. (2014). Diagnosing vulnerability and “dangerousness”: police use of Section 136 in England and Wales. *Journal of Public Mental Health*. 13(2). pp 70-82.
17. Laidlaw, J., Pugh, D., and Maplestone, H. (2009). Section 136 and the psychiatric intensive care unit: setting up a health based place of safety in Gloucestershire. *Journal of Psychiatric Intensive Care*, *5*(2). pp 107-112.
18. Pugh, D. and Laidlaw, J. (2016). Sections 135 and 136: Running a health-based place of safety in Gloucestershire. *Medicine, Science and the Law*, *56*(2), 99-106.
19. Howe, A., Hendry, J., and Potokar, J. (2003). A survey of liaison psychiatry services in the south-west of England. *Psychiatric Bulletin*, *27*(3). pp 90-92.
20. Sondhi, A. et al. (2018). Patient perspectives of being detained under section 136 of the Mental Health Act: Findings from a qualitative study in London. *Medicine, Science and the Law*, *58*(3). pp 159-167.
21. Lowe-Ponsford, F. L., and Begg, A. (1996). Place of safety and section 136 at Gatwick Airport. *Medicine, Science and the Law*, *36*(4). pp 306-312.
22. McClimens A, et al (2017) Evaluation of a mental health liaison team. Part 1: background and literature review. Emergency Nurse. 25(7). pp 31-34.
23. Eales, S., Callaghan, P., and Johnson, B. (2006). Service users and other stakeholders’ evaluation of a liaison mental health service in an accident and emergency department and a general hospital setting. *Journal of psychiatric and mental health nursing*, *13*(1). pp 70-77.
24. Morgan, V., and Coleman, M. (2000). An evaluation of the implementation of a liaison service in an A&E department. *Journal of psychiatric and mental health nursing*, *7*(5). pp 391-397.
25. Callaghan, P. et al (2001). Characteristics of an accident and emergency liaison mental health service in East London. *Journal of Advanced Nursing*, *35*(6). pp 812-818.
26. Ryrie, I., Roberts, M. and Taylor, R. (1997) ‘Liaison psychiatric nursing in an inner city accident and emergency department’, Journal of Psychiatric and Mental Health Nursing, 4, pp. 131–136. doi: 10.1046/j.1365-2850.1997.00031.x.
27. Roberts, D. (1997). Liaison mental health nursing: origins, definition and prospects. *Journal of Advanced Nursing*, *25*(1), 101-108.
28. Ul Haq, S., Ratnayake, T. and Agius, M. (2011) ‘P02-155 - The mental health assessment unit; A novel method for assessing new inpatients’, European Psychiatry. Elsevier, 26, p. 751. doi: 10.1016/S0924-9338(11)72456-7.
29. Agrawal, V. and Murphy, M. (2008) ‘Assessment Unit in psychiatry – a different way of working’, Psychiatric Bulletin, 2 January, p. 33. doi: 10.1192/pb.32.1.33a.
30. Kirubarajan, A. et al (2018). Street triage services in England: service models, national provision and the opinions of police. *BJPsych bulletin*, *42*(6). pp 253-257.
31. Lloyd‐Evans, B. et al (2018). National implementation of a mental health service model: a survey of crisis resolution teams in England. *International journal of mental health nursing*, *27*(1). pp 214-226.
32. Newbigging, K. et al. (2017) ‘Contribution of the voluntary sector to mental health crisis care in England : protocol for a multimethod study’, pp. 1–9. doi: 10.1136/bmjopen-2017-019238.
33. Morant, N. et al. (2017). Crisis resolution and home treatment: stakeholders’ views on critical ingredients and implementation in England. *BMC psychiatry*, *17 (*254). pp 1-13
34. Nelson, L., Miller, P. K. and Ashman, D. (2016) ‘“Dale”: an interpretative phenomenological analysis of a service-user ’ s experience with a Crisis Resolution / Home Treatment team in the UK . Journal of Psychiatric and Mental Health “ Dale ”: An interpretative phenomenological analysis of a service - u’, 23(6–7), pp. 438–448.
35. Lloyd-Evans, B. et al. (2016). Development of a measure of model fidelity for mental health Crisis Resolution Teams. *BMC psychiatry*, *16*(427). pp 1-12
36. Paton, F. et al. 2016). Improving outcomes for people in mental health crisis: a rapid synthesis of the evidence for available models of care. *Health Technology Assessment*, *20*(3). doi: 10.3310/hta20030
37. Morrow, R., McGlennon, D. and McDonnell, C. (2016) ‘A novel mental health crisis service ? outcomes of inpatient data’, Ulster Medical Journal, 85(1), pp. 13–17.
38. Wilson-Palmer, K. and Poole, R. (2015) ‘Street triage for mental health crises.’, British Journal of Nursing, 24(20), pp. 1026–1027.
39. Wheeler, C. et al. (2015) ‘Implementation of the Crisis Resolution Team model in adult mental health settings: A systematic review’, BMC Psychiatry. 15(1), pp. 1–14. doi: 10.1186/s12888-015-0441-x.
40. Loader, K. (2014). Resolving the psychiatric bed crisis: a critical analysis of policy. *British Journal of Nursing*, *23*(3), 150-155.
41. Wise, J. (2014). Emergency departments should not be default option for people with mental health crisis, says report. *BMJ*. 349 doi: <https://doi.org/10.1136/bmj.g6873>
42. Wise, J. (2013). People in mental health crises are treated like criminals, says report. *BMJ*. 346 doi: <https://doi.org/10.1136/bmj.f4036>
43. Sprinks, J. (2014). RCN signs up to'999 plan' aimed at improving delivery of crisis care. *Nursing Standard*. 28(26). p13
44. Duffin, C. (2014). Move to improve emergency care for young people in mental health crisis. *Nursing Children and Young People. 26*(2). p9.
45. Ferguson, B et al. (2010). P02-08-Mental health crisis resolution and home treatment teams: a qualitative evaluation. *European Psychiatry*, *25*(1). pp 626.
46. Sjølie, H., Karlsson, B. and Kim, H. S. (2010) ‘Crisis resolution and home treatment: Structure, process, and outcome - a literature review’, Journal of Psychiatric and Mental Health Nursing, 17(10), pp. 881–892. doi: 10.1111/j.1365-2850.2010.01621.x
47. Julie Morton (2009) Crisis Resolution: A Service Response to Mental Distress, Practice: Social Work in Action, 21(3). pp 143-158, DOI: 10.1080/09503150902807599
48. Morton, J. (2010). Emotion in crisis: Primary and secondary mental health contexts. *Journal of Social Work Practice*, *24*(4). pp 461-474.
49. Johnson, S., et al. (2010). Alternatives to standard acute in-patient care in England: roles and populations served. *The British Journal of Psychiatry*, *197*(S53). pp 6-13.
50. Johnson, S. et al (2009). In-patient and residential alternatives to standard acute psychiatric wards in England. *The British Journal of Psychiatry*, *194*(5). pp 456-463.
51. Lyons, C. et al. (2009). Mental health crisis and respite services: service user and carer aspirations. *Journal of Psychiatric and Mental Health Nursing*, *16*(5). pp 424-433.
52. Khalifeh, H. et al (2009). Home treatment as an alternative to hospital admission for mothers in a mental health crisis: a qualitative study. *Psychiatric Services*, *60*(5). pp 634-639.
53. Howard, L.M., Rigon, E., Cole, L., Lawlor, C. and Johnson, S., (2008) Admission to women's crisis houses or to psychiatric wards: women's pathways to admission. *Psychiatric Services*, 59(12), pp.1443-1449.
54. Hopkins, C., and Niemiec, S. (2006). The development of an evaluation questionnaire for the Newcastle Crisis Assessment and Home Treatment Service: finding a way to include the voices of service users. *Journal of psychiatric and mental health nursing*, *13*(1). pp 40-47.
55. Hopkins, C., and Niemiec, S. (2007). Mental health crisis at home: service user perspectives on what helps and what hinders. *Journal of psychiatric and mental health nursing*, *14*(3). pp 310-318.
56. Johnson, S., Nolan, F., Pilling, S., Sandor, A., Hoult, J., McKenzie, N., White, I.R., Thompson, M. and Bebbington, P., (2005). Randomised controlled trial of acute mental health care by a crisis resolution team: the north Islington crisis study. *BMJ*, 331(7517), p.599.
57. Dunn, L. M. (2001). Mental health act assessments: does a community treatment team make a difference?. *International journal of social psychiatry*, *47*(2), 1-19.
58. Pullen, I. M., and Nisbet, E. (2001). A second survey of Scottish emergency psychiatry. *Health bulletin*, *59*(4). pp 228-232.
59. Kehoe, R. F. and Pullen, I. M. (1992). Emergency psychiatry: a neglected area of a Cinderella service?. *Irish Journal of Psychological Medicine*, *9*(2), pp 105-107.
60. Killaspy, H., Dalton, J., Mcnicholas, S., & Johnson, S. (2000). Drayton Park, an alternative to hospital admission for women in acute mental health crisis. *Psychiatric Bulletin*, *24*(3), 101-104.
61. Killaspy, H. et al. (2000). Drayton Park, an alternative to hospital admission for women in acute mental health crisis. *Psychiatric Bulletin*, *24*(3). pp 101-104.
62. Reed, A. (1998). Manufacturing a human drama from a psychiatric crisis: crisis intervention, family therapy and the work of RD Scott. *Journal of psychiatric and mental health nursing*, *5*(5). pp 387-392.
63. Huxley, P and Kerfoot, M. (1995) letter from Manchester: a typology pf crisis services for mental health. *Journal of Mental Health*. 4. pp 431-435
64. Tufnell, G et al. (1985). Home assessment and treatment in a community psychiatric service. *Acta Psychiatrica Scandinavica*, *72*(1). pp 20-28.
65. Macaskill, A. M., Brodie, B. A. and Keil, B. (2011) ‘Scottish place of safety legislation: local audit of Section 297 Mental Health (Care and Treatment) (Scotland) Act 2003’, The Psychiatrist. Cambridge University Press, 35(05), pp. 185–189. doi: 10.1192/pb.bp.110.030874.
66. Trethewey S. P, Deepak S, Saad S, Hughes, E. and Tadros, G. (2019) Evaluation of the Psychiatric Decisions Unit (PDU): effect on emergency department presentations and psychiatric inpatient admissions. *Postgrad Med Journal*. 95. pp 6–11
67. Hughes et al (2017) *An evaluation of the Psychiatric Decisions Unit (PDU) and its role within the urgent care pathway in Birmingham*. Poster presentation. Birmingham and Solihull Mental Health NHS Foundation Trust.
